# Utilization of Ogura CMS germplasm with the clubroot resistance gene by fertility restoration and cytoplasm replacement in *Brassica oleracea* L

**DOI:** 10.1038/s41438-020-0282-8

**Published:** 2020-05-01

**Authors:** Wenjing Ren, Zhiyuan Li, Fengqing Han, Bin Zhang, Xing Li, Zhiyuan Fang, Limei Yang, Mu Zhuang, Honghao Lv, Yumei Liu, Yong Wang, Hailong Yu, Yangyong Zhang

**Affiliations:** Institute of Vegetables and Flowers, Chinese Academy of Agricultural Sciences, Key Laboratory of Biology and Genetic Improvement of Horticultural Crops, Ministry of Agriculture, #12 Zhong Guan Cun Nandajie Street, Beijing, 100081 China

**Keywords:** Plant breeding, Agricultural genetics

## Abstract

Clubroot disease, a major plant root disease caused by *Plasmodiophora brassicae*, has become one of the most destructive diseases among cultivated cruciferous vegetables. However, clubroot-resistant *Brassica oleracea* materials are rare. A few clubroot-resistant cabbage varieties are available on the market, but all are Ogura cytoplasmic male sterile (CMS) types. Therefore, in this study, to reutilize the clubroot-resistant Ogura CMS germplasm of cabbage, a new fertility-restored Ogura CMS material, 16Q2-11, was used as a bridge to transfer the clubroot resistance (CR) gene from the Ogura CMS cytoplasm to the normal cytoplasm by a two-step method (a fertility restoration and cytoplasm replacement method). In the first cross for fertility restoration of Ogura CMS clubroot-resistant cabbage (FRCRC), 16Q2-11 was used as a restorer to cross with Ogura CMS materials containing the CR gene *CRb2*. Eleven *Rfo*-positive progenies were generated, of which four contained *CRb2*: F8-514, F8-620, F8-732 and F8-839. After inoculation with race 4 of *P. brassicae*, these four *CRb2*-positive individuals showed resistance. Furthermore, F8-514 and F8-839 were then used as male parents in the second cross of FRCRC to cross with cabbage inbred lines, resulting in the successful introgression of the *CRb2* gene into the inbred lines. All offspring produced from this step of cross, which had a normal cytoplasm, showed a high resistance to race 4 of *P. brassicae* and could be utilized for the breeding of clubroot-resistant cabbage varieties in the future. This is the first time that the Ogura CMS restorer has been used to restore the fertility of Ogura CMS clubroot-resistant cabbages, which could improve germplasm diversity in cabbage and provide a reference method for using CMS germplasm in *Brassica* crops.

## Introduction

Cabbages and other brassicas are widely cultivated, with a global harvest area of 2.513 million ha^1^, and play important roles in year-round supplies and exports of vegetables. Clubroot disease of cruciferous vegetables, caused by *Plasmodiophora brassicae*, is a globally distributed, destructive disease. After infection by clubroot pathogens, the roots of plants are often deformed and galled, resulting in reduced or no yield. Moreover, prevention and control of clubroot disease are very difficult. The pathogens can survive in the soil for more than eight years, and the dormant spores can live for more than 20 years in the soil without infecting a host plant^2^; thus, to prevent further infection, infected areas cannot be used for the subsequent cultivation of cruciferous crops for a long time. In light of the limited effectiveness of fungicides and the environmental pollution associated with fungicide use, the development of clubroot-resistant varieties is the most economical and effective way to control clubroot disease^3^. In *Brassica oleracea*, clubroot resistance (CR) is a quantitative trait controlled by multiple genes^4^. Although more than 50 CR quantitative trait loci (QTLs) have been identified^5–14^, it is very difficult to accelerate the breeding process and obtain pure resistant materials because of the complex inheritance of resistance genes. Valuable CR loci have been reported in turnip, Chinese cabbage, radish and rapeseed. To date, at least 19 CR genes/QTLs have been identified in *B. rapa*: *CRa*^15^, *CRb*^16^, *CRb*^*kato*^ ^17^, *CRc*^18^*, CRk*^18^, *CRd*^19^, *CRs*^20^, *Crr1*^21^*, Crr2*^21^, *Crr3*^22^, *Crr4*^21^*, CrrA5*^23^*, Rcr1*^24^, *Rcr2*^25^, *Rcr4*^26^, *Rcr8*^26^, *Rcr9*^26^, *PbBa3.1*^27^ and *PbBa3.3*^27^. *CRb* and *CRa* are recognized as the most fundamental and important genes related to CR. To distinguish the two *CRb* genes, we named them *CRb1*^16^ and *CRb2*^17^ according to the guidelines for gene nomenclature^28^. Researchers have attempted to transfer CR genes from other crucifer crops to *B. oleracea* by distant hybridization^29,30^. Breeders from Syngenta Seeds B·V. produced interspecific crosses between a broccoli inbred line and Chinese cabbage cv. Parkin and then conducted a backcross program with cauliflower, cabbage, and Brussels sprouts to transfer CR genes from the *B. rapa* CR sources. The project entailed several years of backcrosses, selections, line developments, and test crosses^30^. Therefore, transfer of a CR gene by distant hybridization typically requires several years of successive backcrosses to reduce the background of the donor parent. Although a few clubroot-resistant varieties of cabbage are available on the market, almost all are Ogura cytoplasmic male sterile (CMS) varieties with the *orf138* gene and thus cannot be self-pollinated.

Male sterility (MS) in higher plants usually refers to the degeneration or loss of function of male gametes. Since the first case was discovered by the German botanist Joseph Gottlieb Kolreuter in 1763^31^, MS has been reported in more than 600 plant species^32^. Ogura CMS is a naturally occurring mutation found in radish^33^ that has since been transferred into *Brassica* crops (*B. oleracea*^34,35^, *B. napus*^36,37^ and *B. rapa*^38^ and *B. juncea*^39^). In recent years, Ogura CMS hybrids have become increasingly popular because of their high seed purity. However, these hybrids cannot be reutilized because Ogura CMS is maternally inherited, and all of the offspring exhibit MS, which prevents self-pollination and the use of these plants in material innovation. For example, XG336, a clubroot-resistant cabbage hybrid, cannot be reutilized because of its sterile cytoplasm. Creating restorer lines of Ogura CMS in *B. oleracea* is an effective way to solve this problem.

However, there are no natural Ogura CMS restorer lines in *B. oleracea*. The restorer gene (*Rfo*) can only be introduced from related species. Yu et al.^40^ successfully introduced the *Rfo* gene from *B. napus* Y403 into *B. oleracea* var. *alboglabra* Y101 by distant hybridization. BC_2_ progenies were produced using a hexaploid strategy^41^. However, these progenies were hardly applicable because of their allopolyploid background. After two generations of backcrossing with Chinese kale and marker-assisted selection (MAS), a fertility-restored material (16Q2-11) with 18 chromosomes using a triploid strategy was successfully created. The morphology of 16Q2-11 was very similar to that of the female parent, Chinese kale, and the material showed good fertility throughout the flowering period, with pollen viability ranging from 60% to 90% (unpublished). These observations suggest that 16Q2-11 could potentially be used as the male parent to restore the fertility of Ogura CMS cabbage.

In this study, clubroot-resistant cabbage germplasms were screened with linkage markers for the *CRb2* (*CRa)* gene, and the *CRb2*-positive germplasms were then screened with an *orf138*-specific marker to determine whether the cytoplasm was Ogura CMS. The *CRb2*-positive Ogura CMS germplasms were then crossed with 16Q2-11 to restore their fertility. Based on MAS, fertility observation and CR identification, individuals with good fertility and CR were selected as male parents and used in crosses with cabbage inbred lines (female). The derived offspring had a normal cytoplasm and the CR locus. The results of this study could facilitate CR breeding and germplasm reutilization in cabbage.

## Materials and methods

### Plant materials and growth conditions

Twenty cabbage hybrids with different levels of resistance to clubroot disease were collected on the market, and 144 additional cabbage inbred lines were created by the Institute of Vegetables and Flowers (IVF), Chinese Academy of Agricultural Sciences (CAAS). Fertility-restored material (16Q2-11) was created by Yu^40^, which carries the *Rfo* gene and exhibits good pollen viability. To replace the Ogura CMS cytoplasm, three elite cabbage inbred lines (2154, 2156, and 16Q140) were used as female parents in the second cross of FRCRC. All the plants were grown in a greenhouse in autumn at the Institute of Vegetables and Flowers (IVF), Chinese Academy of Agricultural Sciences (CAAS).

The pathogen *P. brassicae* used in this study was collected from Wulong, Chongqing, China, and identified as race 4 based on Williams’ differential system. The cabbage variety Jingfeng No. 1 was used as the susceptible control for CR identification.

### DNA extraction and PCR amplification

Plant genomic DNA was extracted from young leaves by a modified cetyltrimethylammonium bromide (CTAB) method^42^. The DNA concentration was measured by a NanoDrop ND-100 spectrophotometer (Thermo Fisher Scientific, Wilmington, DE, USA), and the DNA was diluted to a working concentration of 40–60 ng μl^−1^ before being stored at −20 °C. The polymerase chain reaction (PCR) mixture (10 μl) contained 1 μl of 10× buffer (containing Mg^2+^), 0.8 μl of dNTPs (2.5 mmol L^−1^), 0.4 µl of forward primers, 0.4 µl of reverse primers, 0.1 μl of Taq DNA polymerase (5 U μl^−1^), 2 μl of template DNA (40–60 ng μl^−1^) and 5.3 μl of distilled H_2_O. The PCR conditions were as follows: predenaturation at 94 °C for 5 min; 35 cycles of denaturation at 94 °C for 30 s, annealing at 58 °C for 30 s, and extension at 72 °C for 45 s; final extension at 72 °C for 5 min; and holding at 4 °C.

A *CRb2*-specific marker, KBrH129J18F/KBrH129J18R^17^, was used to detect the CR locus *CRb2*, and PCR amplicons were separated by 8% polyacrylamide gel electrophoresis at 160 V for 75 min, followed by silver staining. The Ogura cytoplasm-specific marker (Bo138F/Bo138R^43^) was used to screen Ogura CMS types, and *Rfo*-specific markers (Bo*Rfo*-2F/Bo*Rfo*-2R and Bo*Rfo*-6F/Bo*Rfo*-6R) were used to detect the fertility restorer gene *Rfo*^40^. PCR amplicons of these three markers were separated on 1.2% agarose gels in 1x Tris-borate-ethylenediaminetetraacetic acid (TBE) buffer and visualized under ultraviolet (UV) light. Information of all markers used in this study is provided in Table S1.

### Fertility restoration of clubroot-resistant Ogura CMS germplasms

The fertility restoration and MAS procedure are shown in Fig. S1. All crosses were performed by artificial pollination. The pollinated flower buds, siliques and harvested seeds were counted to calculate the number of seeds per silique.

The progenies were then screened with *CRb2*- and *Rfo-*specific markers. The fertility-related characteristics, including pollen viability and pollen amount, of the *CRb2*- and *Rfo*-positive individuals were measured. Pollen viability was determined at the flowering stage by the aceto-carmine dyeing method. Pollen from three newly opened flowers on each plant was spread onto slides and then stained with 1% acetocarmine. The average number of viable pollen grains in three replicates was calculated. More than 300 pollen grains were observed under the microscope for each replicate. Viable pollen was plump and deep pink. The amount of pollen was confirmed with the naked eye, and the maintainer line of the Chinese kale parent Y101 was used as a control (100%) to divide the pollen amounts into five grades: grade 5, 80–100%; grade 4, 60–80%; grade 3, 40–60%; grade 2, 20–40%; and grade 1,0–20%.

### Morphological observation and ploidy analysis of fertility-restored clubroot-resistant individuals

Morphological characteristics, including plant type, leaf shape, leaf color, leaf surface wax, bud traits, inflorescence traits, and progeny flower color, were investigated according to the standards described in “Descriptors and data standards for Chinese kale”^44^.

Ploidy identification was performed using flow cytometry (FCM) (BD FACSCalibur, BD Biosciences, San Jose, CA, USA). Sample preparation followed the method of Dolezel^45^, with some modifications: (1) a piece of fresh, young cabbage leaf was placed in a Petri dish, its veins were removed, and 2 ml of ice-cold Galbraith’s nucleus isolation buffer was added to the Petri dish; (2) a sharp scalpel was used to quickly cut the leaf and free the nuclei; (3) the solution in the culture dish was aspirated and filtered through a 37-mm nylon mesh into a 1.5-ml centrifuge tube; and (4) 500 μl of propidium iodide (PI) stock solution was added to the centrifuge tube, which was then incubated in ice for 30 min. The solution was mixed by shaking the centrifuge tube before FCM analysis. The Chinese kale parental DNA content (2 C) was used as the reference, and its G1 phase peak was positioned on the abscissa in 200 channels. The relative cellular DNA content of the treatment samples was determined if the coefficient of variation (CV) was below 5%.

### Identification of CR in CRb2-positive individuals

The clubroot disease resistance of *CRb2*-positive individuals was identified by artificial inoculation, with three repeats and four plants per repeat. The resting spore inoculum was prepared by a modified protocol^46^. The artificial inoculation method was as follows: (1) the clubbed roots were thawed, combined with a triple volume of distilled water, and homogenized in a blender; (2) the slurry was passed through eight layers of cheesecloth, and the suspension was centrifuged with a freezing centrifuge at 500 *× g* for 5 min; (3) the precipitate was discarded, and the supernatant was transferred to a new tube and centrifuged at 2000 *× g* for 5 min; (4) distilled water was used to wash the resulting pellet three times; (5) the surface of the pellet was disinfected by incubation with colistin sulfate and vancomycin hydrochloride (1 mg ml^−1^, Sigma-Aldrich, Canada) in distilled water at 25 °C in the dark for 24 h; (6) the suspension was washed in sterile water twice after centrifugation; (7) a hemocytometer was used to count the resting spores, and the resulting values were adjusted to 2 × 10^8^ spores ml^−1^; (8) the suspension was stored at 4 °C and used within 24 h; (9) at the two-real-leaf stage, blades were used to cut the seedlings’ roots before inoculation; and (10) 5 ml of prepared resting spore suspension was injected into the bottom of the stem of each seedling in the soil using a transferpettor.

Disease resistance was evaluated 50 days after inoculation (DAI). The classification standard of CR^47^ was applied as follows: grade 0, normal root growth and no visible root galls; grade 1, normal growth of the main root and several small galls on the lateral and fibrous roots; grade 2, mild galls on the taproot and many small or large galls on the lateral and fibrous roots; grade 3, severe galls on the taproot and galls present on most of the lateral and fibrous roots; and grade 4, almost no lateral roots or fibrous roots on the plant, taproot abnormally enlarged, and no healthy roots present. The disease index (DI) was calculated as DI = [∑ab/cd] × 100, where a is the number of plants of each grade, b is the corresponding disease symptoms (0–4), c is the total number of plants tested, and d is the highest grade. The resistance evaluation criterion was formulated by the National Key Cabbage Breeding Technologies R&D Program of China during the 9th 5-year Plan period, where DI = 0 corresponds to immunity (I), 0 < DI ≤ 5 corresponds to high resistance (HR), 5 < DI ≤ 20 corresponds to resistance (R), 20 < DI ≤ 30 corresponds to medium resistance (MR), 30 < DI ≤ 60 corresponds to susceptibility (S), and DI > 60 corresponds to high susceptibility (HS).

## Results

### Screening of clubroot-resistant cabbage germplasms

**Fig. 1:**
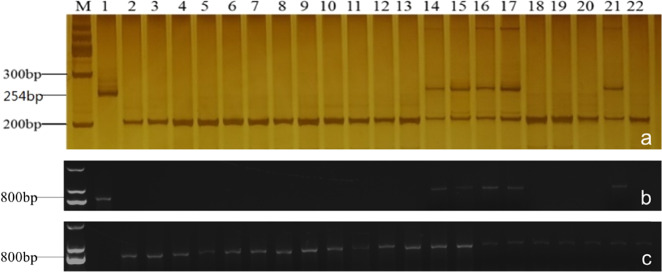
PCR amplification using the *CRb2*-specific marker KBrH129J18F/KBrH129J18R (**a**) and the *CRa*-specific markers SC2930-T-FW/SC2930-RV (**b**) and SC2930-Q-FW/SC2930-RV (**c**) in 20 Ogura CMS cabbage hybrid resources. 1: resistant control (CK) of Chinese cabbage, 2: susceptible CK, 3-22: 20 Ogura CMS cabbage materials. Materials 14, 15, 16, 17, and 21 (corresponding to germplasms 2161, 2169, 2171, 2172, and 2173) harbored the *CRb2* locus

All 20 cabbage germplasms were assessed for resistance to clubroot disease by artificial inoculation. Five of the germplasms (2161, 2169, 2171, 2172, and 2173) showed high resistance to race 4 of *P. brassicae* (Table S2). Fourteen pairs of clubroot-resistant primers for the detection of *CRa*^48,49^*, Cram*^49^*, CRb1*^16,50^*, CRb2*^17,51^*, CRc*^48^*, Crr1*^52^*, Crr2*^21^, and *Crr3*^21^ were used to screen the 20 cabbage germplasms (Table S1). Only the *CRb2*-specific marker KBrH129J18 and *CRa*-specific marker SC2930 amplified CR-associated fragments in 2161, 2169, 2171, 2172, and 2173, which was consistent with the inoculation results. The size of the fragments was the same as that of clubroot-resistant Chinese cabbage (CK). Furthermore, all five materials were heterozygous at the *CRb2* locus (Fig. 1). However, all five materials were male sterile. Screening by the *Orf138*-specific marker revealed that they contained the Ogura CMS cytoplasm (Fig. S2). Therefore, all five clubroot-resistant cabbage germplasms with the *CRb2* locus exhibited CMS, which prevented self-pollination and their use in germplasm innovation.

Detection of CR by artificial inoculation and molecular markers was conducted for 144 additional cabbage inbred lines, but no resistant lines were identified. Overall, among the 164 materials (144 inbred lines and 20 germplasms) used in this study, clubroot-resistant germplasms with the *CRb2* locus in cabbage were available only in the Ogura CMS materials, which could be reutilized if their fertility was recovered.

### Fertility recovery of clubroot-resistant Ogura CMS cabbages by 16Q2-11

The fertility-restored line 16Q2-11 was used as the male parent in the first FRCRC cross with five Ogura CMS germplasms containing the *CRb2* locus (2161, 2169, 2171, 2172, and 2173). Pollination of 1480 flower buds in the five crosses was conducted, resulting in the production of 962 siliques and 3872 seeds. The seed setting rate of the five crosses ranged from 3.18 to 5.33 seeds pod^−1^ (Table 1). Significant differences in the seed setting rate were observed among the five combinations (*P* < 0.05), and the highest seed setting rate, 5.33 seeds pod^−1^, was exhibited by the combination 2161 × 16Q2-11 (Table 1).

**Table 1:** Seed setting results for five cross combinations between five clubroot-resistant Ogura CMS cabbages and 16Q2-11 in the first FRCRC cross

Female parent	Number of pollinated flowers	Number of siliques	Number of seeds	Seeds per pod (mean ± SD, SP)	Number of viable plants	Number of *Rfo*-positive individuals	Transmission rate of *Rfo* (%)
2161	212	75	402	5.33^aA^ ± 0.062	366	1	0.27
2169	342	208	850	4.09^dC^ ± 0.037	796	0	0
2171	267	204	900	4.37^cB^ ± 0.028	836	5	0.60
2172	455	320	1020	3.18^eD^ ± 0.010	948	5	0.53
2173	204	155	700	4.52^bB^ ± 0.010	674	0	0

Uppercase letters indicate extremely significant differences (*P* < 0.01), and lowercase letters indicate significant differences (*P* < 0.05).

**Fig. 2:**
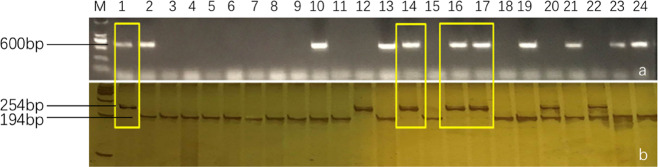
Screening of *Rfo* (**a**) and *CRb2* (**b**) in hybrids between five clubroot-resistant Ogura CMS cabbage materials and 16Q2-11 in the first FRCRC cross. Materials 1, 14, 16, and 17 were Ogura CMS cabbages containing both the *Rfo* and *CRb2* loci. FRCRC denotes fertility restoration of Ogura CMS clubroot-resistant cabbages

An *Rfo*-specific marker was used to screen all the progenies derived from crosses between 16Q2-11 and *CRb2*-positive Ogura CMS cabbage germplasms, and only 11 plants were *Rfo* positive (Table 1, Fig. 2a). All 11 *Rfo*-positive plants were Ogura CMS (Fig. S3). Among the crosses, the combination 2171 × 16Q2-11 produced the largest proportion of *Rfo*-positive plants; in contrast, the combinations 2169 × 16Q2-11 and 2173 × 16Q2-11 produced no *Rfo*-positive plants (Table 1). The transmission rates (TRs) of *Rfo* for the five combinations 2161 × 16Q2-11, 2169 × 16Q2-11, 2171 × 16Q2-11, 2172 × 16Q2-11, and 2173 × 16Q2-11 were 0.27%, 0%, 0.60%, 0.53%, and 0%, respectively.

**Fig. 3:**
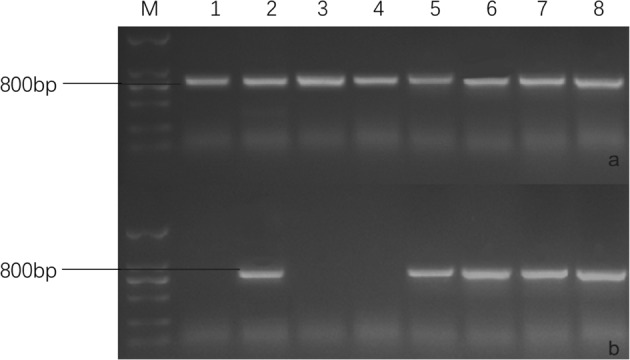
PCR amplification using the *CRa*-specific markers SC2930-Q-FW/SC2930-RV (**a**) and SC2930-T-FW/SC2930-RV (**b**). 1: 16Q2-11, 2: 2161, 3: Y101, 4: Y403, 5: F8-514, 6: F8-620, 7: F8-732, 8: F8-839

Among the 11 *Rfo*-positive plants, four individuals (F8-514, F8-620, F8-732, and F8-839) contained *CRb2* (*CRa*) (Figs. 2b and 3). The TR of *CRb2* in the first FRCRC cross was 36.4% (4/11). F8-514 and F8-620 were obtained from the combination 2171 × 16Q2-11, and F8-732 and F8-839 were obtained from the combination 2172 × 16Q2-11.

During the flowering period, the pollen amounts were categorized; those of F8-514 and F8-839 were all categorized as grade 5, whereas those of F8-620 and F8-732 were categorized as grades 1 and 2, respectively (Fig. S4a–d). The average number of viable pollen grains across three replicates per plant was calculated. The pollen viabilities of F8-514 and F8-839 were >80%, whereas those of F8-620 and F8-732 were <40% (Fig. S4e–h).

### Morphology, ploidy and CR of the four Rfo- and CRb2-positive plants

The morphological characteristics of the four *Rfo-* and *CRb2*-positive plants were investigated. Most traits, including plant type, flower size, and leaf type, exhibited intermediate phenotypes between those of Chinese kale and cabbage, and the plants showed strong growth. However, some morphological characteristics were more similar to those of a parent. For instance, the flower color was white, similar to that of Chinese kale, whereas the leaves were gray-green, similar to those of cabbage (Fig. 5a, b).

Ploidy analysis revealed that all four plants were diploid, which is consistent with the ploidy of cabbage and Chinese kale (Fig. S5c). All *Rfo*- and *CRb2*-positive plants grew normally and showed no obvious disease symptoms after inoculation with *P. brassicae* race 4, whereas the susceptible control grew slowly and formed clubbed roots (Fig. S5d, e). Therefore, considering CR and pollen viability, F8-514 and F8-839 were chosen for further backcrossing.

### Creation of clubroot-resistant germplasms with a normal cabbage cytoplasm

Because F8-514 and F8-839 contain an Ogura CMS cytoplasm, their self-pollinated offspring also contain this cytoplasm. Therefore, in the second cross of FRCRC, the fertility-restored and clubroot-resistant individuals (F8-514 and F8-839) used as the male parent were crossed with elite inbred lines (2154, 2156, and 16Q140) whose cytoplasm was normal.

In the second FRCRC cross, F8-514 and F8-839 were used as male parents, and three elite cabbage inbred lines (2154, 2156, and 16Q140) were used as female parents. A total of 82 pollinated buds and 65 siliques were obtained from the three cross combinations, and 788 seeds were harvested. The seed setting rate of the three combinations ranged from 10.8 to 12.3 seeds pod^−1^, which was much higher than that observed in 16Q2-11 and showed no difference among the three inbred lines (Table 2). A total of 551 seeds were sown, and 536 seedlings were obtained.

**Table 2:** Seed setting results for three crosses between nine cabbage inbred lines and F8-839 and F8-514 in the second FRCRC cross

Female parent	Male parent	Number of pollinated flowers	Number of siliques	Number of seeds	Seeds per pod	Number of viable plants	Number of individual plants containing the *CRb2* resistance locus	Expected ratio	χ
2154	F8-514	28	22	271	12.3	72	32	1:1	0.44
2156	F8-514	47	38	463	12.2	392	183	1:1	0.86
16Q140	F8-839	7	5	54	10.8	72	30	1:1	1

**Fig. 4:**
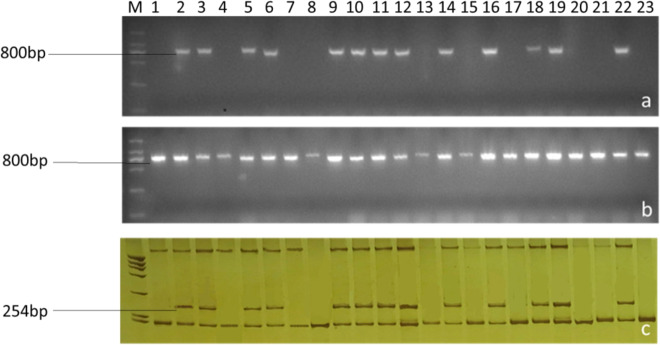
Screening of the *CRa* (*CRb2*) locus in hybrid progenies of the second FRCRC cross. **a** PCR amplification of SC2930-T-FW/SC2930-RV, **b** PCR amplification of SC2930-Q-FW/SC2930-RV, **c** PCR amplification of KBrH129J18-F/KBrH129J18-R

All progenies were screened with *CRb2*- and *CRa*-specific markers (Fig. 4). There were 245 plants containing the *CRb2* (*CRa*) locus in the three populations derived from the crosses 2154 × F8-514, 2156 × F8-514 and 16Q140 × F8-839 (Table 2), which was confirmed by a 1:1 segregation ratio at the *CRb2* locus by chi-square test. Furthermore, their fertility showed no significant differences from that of normal plants (the elite cabbage inbred lines).

Bo138F/Bo138R marker screening revealed that all the progenies had a normal cytoplasm, indicating that the *CRb2* locus had been successfully introgressed into cabbage with a normal cytoplasm.

**Fig. 5:**
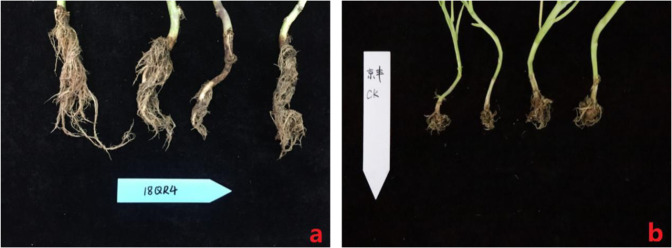
Identification of clubroot disease resistance in 18QR4 (**a**) and the susceptible control (CK) Jingfeng (**b**) after inoculation with race 4 of *P. brassicae*

Twelve *CRb2-*containing plants of 18QR4 (from cross 2156 × F8-514) were randomly selected to identify their resistance to clubroot disease. Eleven of the 12 plants of 18QR4 were assigned a grade of 0, showing high resistance to clubroot disease, and only one plant was assigned a grade of 1, which had a few small clubs on the lateral root (Fig. 5a). The DI of 18QR4 was 2.08, corresponding to HR. The susceptible control Jingfeng No. 1 was highly susceptible to clubroot disease (Fig. 5b).

## Discussion

### Significance of the bridge material 16Q2-11

Clubroot disease reduces both the quality and yield of cruciferous crops, posing a serious threat to the production of these crops worldwide^53,54^. The breeding of clubroot-resistant varieties is the most economical and effective method for controlling clubroot disease. CR genes have been reported in many germplasm resources of *B. rapa*^15–27^ but are very rare in *B. oleracea*. To date, many Chinese cabbage and rapeseed varieties with CR have been successfully cultivated and entered into the market, but few CR cabbage varieties can be found on the market^55^. Crisp et al.^56^ screened and analyzed a total of 1047 (2*n* = 18, CC) accessions of *B. oleracea*, of which only 7.4% possessed resistance to two highly virulent isolates of *P. brassicae* from the United Kingdom. Similarly, Ning et al.^57^ in our group collected 102 cabbage genotypes and evaluated them for resistance to *P. brassicae* race 4, the predominant race in China; only one highly resistant genotype, XG336, was identified that has the potential to serve as a resistant source for the breeding of clubroot-resistant cabbage. However, XG336 cannot be reutilized because of its Ogura cytoplasm. Similarly, in our study, we also obtained five materials that have high resistance to *P. brassicae* race 4 among 164 cabbage germplasms (20 clubroot-resistant cabbage germplasms and 144 additional cabbage inbred lines). All of them were Ogura CMS materials screened by the Ogura CMS marker. Our study, together with other reports, highlights the importance of a restorer for Ogura CMS cabbages. To re-utilize the Ogura clubroot-resistant cabbage germplasms, in our study, a fertility-restored Ogura CMS line (16Q2-11), which was created in previous research, was successfully used as a bridge to transfer CR into normal-cytoplasm lines. In this study, we found that the TR of the *Rfo* gene was <1%. The introgression of alien chromosomal fragments in 16Q2-11, which might result in disordered or abnormal chromosome pairing/segregation, was the main reason for the phenomena. The importance of the bridge material 16Q2-11 is significant, as once the fertility of Ogura CMS material is restored and the separation rate of *CRb2* is normal, the reutilization of Ogura cytoplasmic CR germplasm is possible. Ogura CMS has been widely used in cabbage, broccoli, cauliflower and many other *Brassica* vegetables because of its stable sterility and ease of transfer^58–60^, which means that a bridge material and the two-step method will have broad application prospects.

### Possible sources of CR conferred by the CRb2 locus in B. oleracea

The genus *Brassica* comprises 38 species and numerous subspecies. Six species compose the “Triangle of U”: the diploids *Brassica rapa* (AA), *Brassica nigra* (BB), and *Brassica oleracea* (CC) and the allotetraploid species *Brassica juncea* (AABB), *Brassica napus* (AACC), and *Brassica carinata* (BBCC). Cheng et al.^61^ analyzed the *B. rapa* ancestral genomes with other *Brassicaceae* genomes; the results suggested that the three *Brassica* genomes (A, B, and C) descended from a common hexaploid ancestor that originated from the merger of three tPCK-like ancestral genomes. Interestingly, many CR genes have been cloned and applied in *B. rapa*, whereas few CR genes have been cloned and applied in *B. oleracea* to date. We found that the *CRb2* gene in *B. rapa* and the *Bo7g107730* gene in *B. oleracea* are homologous; we speculated that the *CRb2* locus in *B. rapa* was domesticated to be functional during natural selection.

**Fig. 6:**
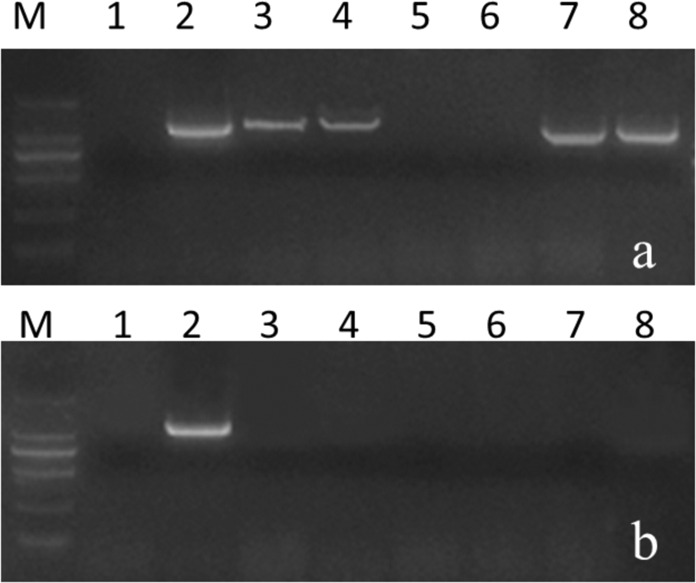
Screening of the *CRa* locus by using marker FW4/RV4. **a** Results of identification for the *CRa* locus; 1: 2160, 2: 2161, 3: 2169, 4: 2171, 5: 2162, 6: 2163, 7: 2172, 8: 2173; **b** partial identification results for the *CRa* locus in 144 cabbage varieties; 1: negative control 2160, 2: positive control 2161

There are two possible explanations for the presence of CR in the *B. oleracea* materials used in this study. One possible explanation is that the resistance locus in these five cabbages is similar to the *CRb2* resistance locus in *B. rapa*, which was domesticated to be functional in the natural selection process. The other possible explanation is that the resistance locus in these five cabbages derives from distant hybridization with Chinese cabbage. Diederichsen et al.^30^ reported research that introduced the CR locus from *B. rapa* to *B. oleracea* by distant hybridization combined with embryo rescue. In the present study, we screened 20 clubroot-resistant cabbage germplasms and 144 additional cabbage inbred lines by using A-genome markers (linkage markers KBrH129J18F/KBrH129J18R and SC2930 and the intragenic marker FW4/RV4, Fig. 6). We found that these three markers amplified corresponding fragments in five clubroot-resistant cabbage varieties; no bands were produced for the 144 cabbage inbred lines, which was consistent with the inoculation results. Thus, we suggest that the resistance *CRb2* locus in *B. oleracea* may be derived from *B. rapa*. In this study, the *CRb2* gene was successfully introgressed into inbred lines with normal cytoplasm with a 1:1 separation ratio by the use of the restorer 16Q2-11 (Table 2), and these lines exhibited high resistance to race 4 of *P. brassicae* (Fig. 5).

These results above indicate that the Ogura CMS restorer created here could be applied to recover the fertility of the valuable Ogura CMS germplasm, thereby potentially facilitating cabbage breeding and helping to increase the diversity of cabbage germplasms. The presented approach could not only provide clubroot-resistant materials for cabbage breeding but also represents a practicable method for the utilization of Ogura CMS resources in the genus *Brassica*.

## Supplementary information

Table S1 and Table S2

Supplementary Figures
