# Supplementary material for: Utilization of Ogura CMS germplasm with the clubroot resistance gene by fertility restoration and cytoplasm replacement in Brassica oleracea L
Source: Hortic Res. 2020 May 1;7:61. doi: 10.1038/s41438-020-0282-8 (PMC7193625; doi:10.1038/s41438-020-0282-8)
Supplement: Supplementary file 2 — Supplementary Figures [file 41438_2020_282_MOESM2_ESM.docx]

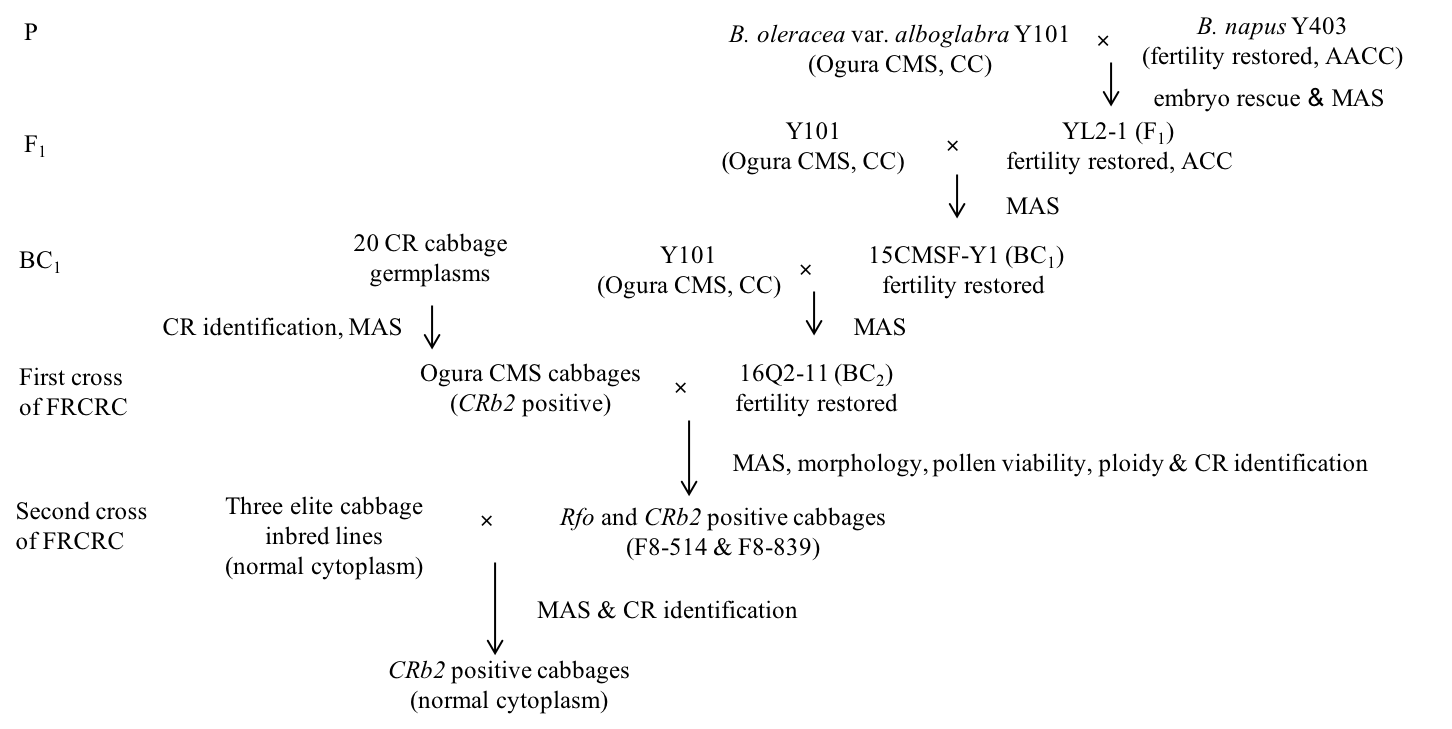


Fig. S1 Breeding process for creating clubroot-resistant cabbage materials by using the Ogura CMS restorer.


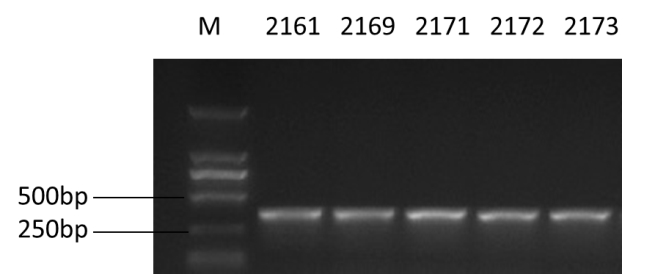


Fig. S2 PCR amplification of five *CRb2*-positive cabbage resources using the marker Bo138F/Bo138R.


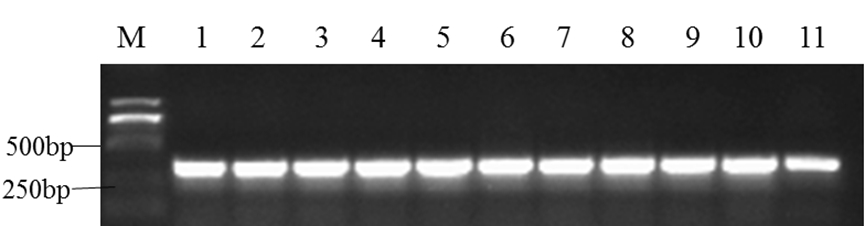


Fig. S3 PCR amplification of 11 *Rfo*-positive cabbage materials using the marker Bo138F/Bo138R.


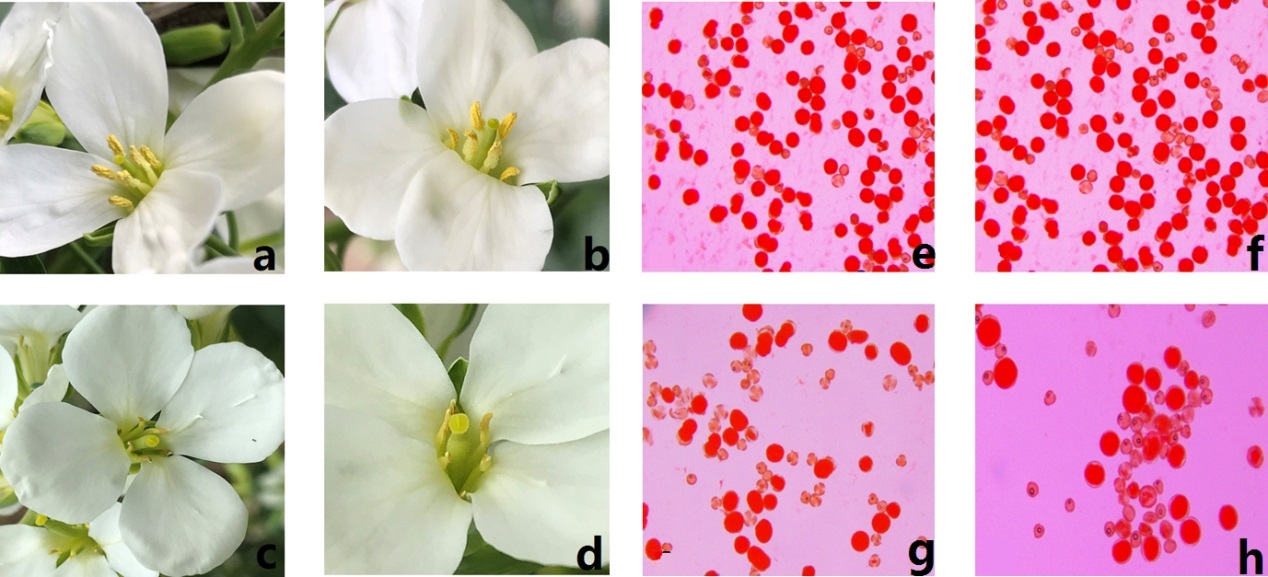
 Fig. S4 Relative pollen grain content in F8-514 (a), F8-839 (b), F8-620 (c) and F8-732 (d). Pollen viability in F8-514 (e), F8-839 (f), F8-620 (g) and F8-732 (h).


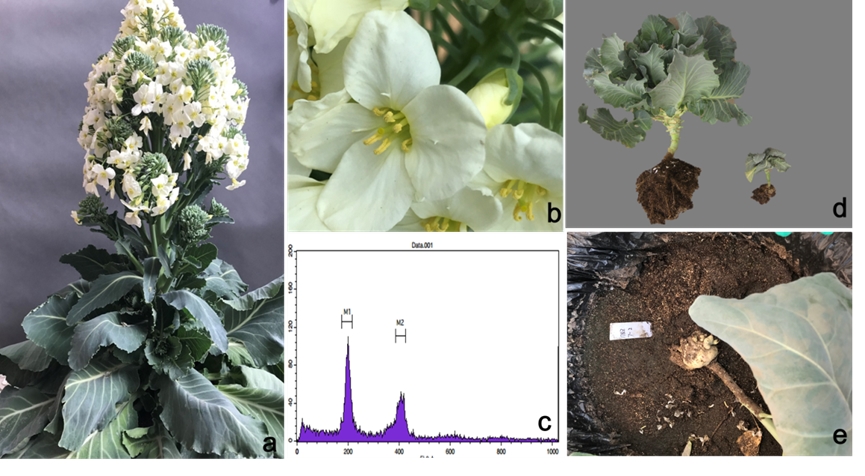


Fig. S5 General morphology (a), flower color (b), ploidy (c) and CR identification based on artificial inoculation (d, e).
